# Current High-Throughput Approaches of Screening Modulatory Effects of Xenobiotics on Cytochrome P450 (CYP) Enzymes

**DOI:** 10.3390/ht7040029

**Published:** 2018-09-29

**Authors:** Yee Tze Ung, Chin Eng Ong, Yan Pan

**Affiliations:** 1 Department of Biomedical Science, the University of Nottingham Malaysia Campus, Jalan Broga, Semenyih 43500, Selangor, Malaysia; khyy6uyt@nottingham.edu.my; 2 School of Pharmacy, International Medical University, Bukit Jalil 57000, Wilayah Persekutuan Kuala Lumpur, Malaysia; ceong98@hotmail.com

**Keywords:** high-throughput screening, inhibitory effects, cytochrome P450, in vitro

## Abstract

Cytochrome P450 (CYP) is a critical drug-metabolizing enzyme superfamily. Modulation of CYP enzyme activities has the potential to cause drug–drug/herb interactions. Drug–drug/herb interactions can lead to serious adverse drug reactions (ADRs) or drug failures. Therefore, there is a need to examine the modulatory effects of new drug entities or herbal preparations on a wide range of CYP isoforms. The classic method of quantifying CYP enzyme activities is based on high-performance liquid chromatography (HPLC), which is time- and reagent-consuming. In the past two decades, high-throughput screening methods including fluorescence-based, luminescence-based, and mass-spectrometry-based assays have been developed and widely applied to estimate CYP enzyme activities. In general, these methods are faster and use lower volume of reagents than HPLC. However, each high-throughput method has its own limitations. Investigators may make a selection of these methods based on the available equipment in the laboratory, budget, and enzyme sources supplied. Furthermore, the current high-throughput systems should look into developing a reliable automation mechanism to accomplish ultra-high-throughput screening in the near future.

## 1. Introduction

Cytochrome P450 (CYP) is a superfamily of enzymes, and in this superfamily, enzymes belonging to the families 1, 2, and 3 play a major role in metabolizing a wide spectrum of xenobiotics [1]. Drugs such as one type of xenobiotics undergo processes of absorption, distribution, metabolism, and elimination throughout the human body [2]. The major CYP isoforms involved in drug metabolism include CYP3A4/5, CYP2D6, CYP2C9, CYP1A2, CYP2B6, CYP2C19, CYP2C8, CYP2A6, CYP2E1, and CYP2J2 [3]. The metabolism of xenobiotics including drugs is the key path of detoxification by adding hydrophilic groups to the molecular structures of parent compounds [4]. Drug–drug interactions may occur if one of the drugs inhibits CYP enzyme activity involved in metabolizing the coadministered drug. Alternatively, drug–drug interactions may also occur when coadministered drugs are metabolized by the same CYP enzyme but one drug has a lower specificity. These situations will lead to an elevation in plasma concentration of the coadministered drug (or drug with lower specificity) and potentially may result in adverse drug reactions (ADRs). ADRs are generally unwanted or harmful reactions following the administration of medications [5]. Various factors are responsible for ADRs, and drug–drug interactions contribute to up to 15% of hospitalized geriatric patients experiencing ADRs [6]. Since they are associated with morbidity and mortality, ADRs are one of the most serious clinical issues in relation to the use of drugs [7]. On the other hand, many prodrugs require bioactivation via CYPs to gain pharmacological activities. Nowadays, efforts have been made to generate prodrugs to improve the bioavailability of drugs, to reduce drug toxicities, and to deliver drugs to specific cells or tissues [8]. Under such circumstances, the inhibition of CYP activity by one drug will lead to the prevention of the activation of the coadministered drug (prodrug). Consequently, the patients consuming the two drugs may experience treatment failure [9].

Drug interactions have been considered with reference to the guidelines during the initial stage of drug discovery. High-throughput screening approaches have been indispensable in routine assays using a commercial kit. Patients are given drugs under the supervision of a medical doctor or pharmacist. Nevertheless, even patients who take a prescribed drug can purchase herbs or herbal preparations, including health food, every time without harm and toxicity information. In addition, drug–herb interactions are tolerated by self-imposed responsibilities of the herb (health food) maker. The use of herbal products as alternative medical therapies has been documented extensively globally, and there is an increasing trend of this practice. People believe that herbs are natural and thus have fewer side effects as compared to modern medicines [10]. Nevertheless, herbs contain numerous active constituents, which have the potential to affect CYP enzyme activities. Hence, taking herbal products and modern medicines concurrently may lead to drug–herb interactions. Similarly, a handful of clinical ADRs are reported due to drug–herb interactions [11]. Therefore, it is important to investigate the modulatory effect of drugs, new chemical entities, and herbal products on CYPs in order to reduce ADRs, to improve the treatment efficacy, and to minimize the number of drugs being withdrawn. 

The traditional approaches to the study of CYP enzyme activity are mainly chromatographic-based assays such as high-performance liquid chromatography (HPLC) [12,13,14,15,16]. Briefly, after the assay mixture containing the CYP enzyme, buffer, probe substrate, as well as cofactors is incubated for a certain period of time, the formation of the metabolite is quantified by the HPLC system. The more the metabolite is formed, the more CYP enzyme activity is expected. Although useful, HPLC-based assays normally require elaborate extraction procedures to remove protein and other big molecules, which may block the HPLC system. These assays also require large assay volumes and high enzyme concentrations to reach the limit of detection. Moreover, HPLC-based assays employ relatively long run times for each sample. Since the late 1990s, high-throughput CYP assay approaches have gained great attention. These assays employ a smaller assay volume (<300 μL), and they are able to carry out multiple assays in a 96-well microtiter plate format [17]. As a result, high-throughput detection of enzyme activities is achieved since a higher number of samples can be screened in one sitting within a couple of seconds. For these reasons, both drug and herb (health food) researchers should innovate high-throughput screening. The purpose of this paper is to review the current available high-throughput methods used to study CYP activities. 

## 2. Fluorescence-Based Assay 

In order to establish fluorimetric methods to quantify CYP enzyme activities, the fluorogenic probes should have good aqueous solubility, well metabolite formation, low background fluorescence, high signal-to-noise ratio, or an appropriate excitation wavelength in the UV range for measuring CYP activities. It was proposed that various *O*-alkyl derivatives of resorufin [18], fluorescein [19], 7-hydroxycoumarins [20], 6-hydroxyquinolines [19], as well as 4-methylsulfonyphenyl furanones [21] could be suitable for this purpose. Since most of the fluorogenic probes are not selective, it is advisable to use heterologously expressed individual recombinant CYP enzymes instead of employing liver microsomes or hepatocytes, which contain several CYPs. Alternatively, a selective fluorescent probe such as (3,4-difluorobenzyloxy)-5,5-dimethyl-4-(4-methylsulfonylphenyl)-(5H)-furan-2-one) (DFB) for the CYP3A enzyme should be applied [22]. Table 1 listed the common fluorogenic substrates of CYPs and their respective metabolites as well as excitation/emission (Ex/Em) wavelengths for the detection. 

**Figure 1:**
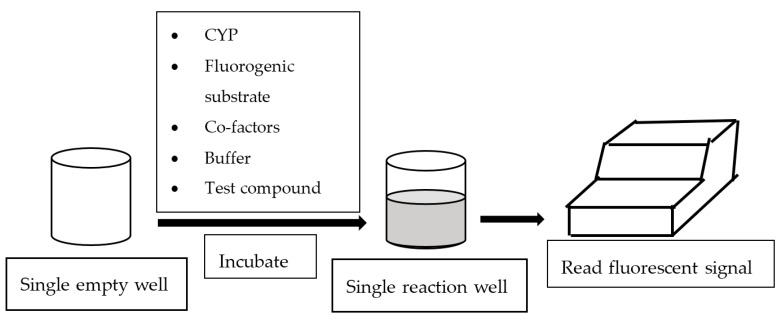
Work flow of fluorescence-based assay.

**Figure 2:**
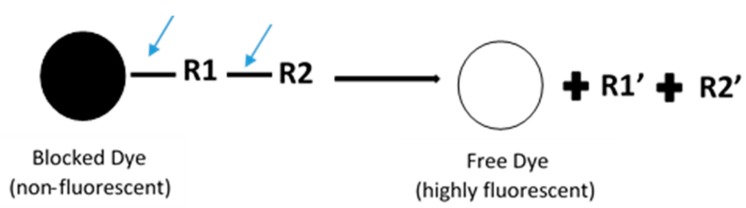
Schematic of the metabolism of the ‘blocked’ dye substrate into a fluorescent metabolite.

In general, fluorescence-based assays carried out in a 96-well plate are performed to screen the inhibitory potencies of a wide range of drugs and herbal constituents. Figure 1 illustrates the general work flow of a fluorescence-based assay performed in a single well. Human recombinant cDNA-expressed CYP3A4 with its nonfluorescent probe substrate 7-benzyloxy-4-(trifluoromethyl) coumarin (BFC) were incubated to investigate the simultaneous inhibition by 11 different compounds (erythromycin, verapamil, ethynilestradiol, miconazole, bromoergocriptine, nicardipine, clotrimazole, roxythromycin, cimetidine, nifedipine, and ketoconazole) [23]. Similarly, IC_50_ values were obtained using recombinant microsomes from baculovirus-infected insect cells, liver CYPs (CYP1A1, CYP1A2, CYP2A6, and CYP3A4) and testing compounds (vorozole and letrozole) employing coumarin, 7-methoxy-4-(trifluoromethyl) coumarin (MFC), 3-cyano-7-ethoxycoumarin (CEC), and BFC as the substrates to screen the inhibitory capability of inhibitors [24]. Moreover, baculovirus/insect cells, cDNA-expressed CYP3A4 using benzyloxyresorufin (BzRes), BFC, 7-benzyloxyquinoline (BQ), and dibenzylfluorescein (DBF) as the fluorometric probe substrates were used to test 27 compounds in inhibition assays [19]. Twenty-nine antiparasitic drugs and positive inhibitors (naphthoflavone, sulfaphenazole, ticlopidine, quinidine, and ketoconazole) were determined by their inhibition of recombinant human CYPs (1A2, 2C9, 2C19, 2D6, and 3A4) expressed from yeast employing CEC, MFC, 7-methoxy-4-(aminomethyl)-coumarin (MAMC), and BFC as substrates [25]. The mechanism-based inhibition of recombinant human c-DNA-expressed CYP2B6 by bergamottin was accessed by using 7-ethoxytrifluoromethyl coumarin (EFC) as the substrate [26]. Nowadays, commercial kits consisting of recombinant CYP, CYP reductase, cofactors, buffer, and fluorogenic substrates (such as BOMCC and EOMCC) have been developed to investigate the inhibitory potencies of compounds on CYP activities. Vivid^®^ CYP450 Screening Kits supplied by ThermoFisher Scientific (Waltham, MA, USA) were widely applied. Inhibitory potentials of standardized extracts of *Tinospora cordifolia* and its bioactive compound on CYP3A4, CYP2D6, CYP2C9, and CYP1A2 were determined using Vivid^®^ CYP450 Screening Kits [27]. Likewise, this kit was also employed to screen the inhibitory effects of *Trigonella foenum-graecum* (TFG) and trigonelline (TG) on several CYP isoforms [28]. Figure 2 demonstrates the scheme of the metabolism of the Vivid^®^ substrate to a fluorescent metabolite. It shows that the substrate is nonfluorescent as its fluorophores are blocked by R1 and R2. The fluorescence signal was triggered after R1 and/or R2 were removed by CYPs.

This fluorescence-based approach has been attached to other systems to achieve ultra-high-throughput, which further accelerated the screening process. Human liver microsomes (CYP1A2, CYP2A6, CYP2B6, CYP2D6, CYP2E1, CYP2C8, CYP2C9, CYP2C19, CYP3A4, and CYP3A5) using DFB as a probe substrate and ketoconazole, miconazole, nicardipine, and nifedipine as the inhibitors for enzyme inhibition assays were carried out through an automated, fluorescent-based, 96-well assay. It was found that DFB was a selective substrate for CYP3A enzymes [21]. An automated, fluorometric, 384-well microplate assay was used to investigate the inhibitory effects of the test compound, leishmania, on human CYP3A4 and CYP2D6 from liver microsomes with BFC and 3-[2-(*N*,*N*-diethyl-*N*-methylammonium)ethyl]-7-methoxy-4-methylcoumarin (AMMC) as substrates [29]. On the other hand, BFC, MFC, 7-methoxy-4-(aminomethyl)coumarin (MAMC), and CEC were employed as substrates for CYP1A2, CYP2C9, CYP2C19, CYP2D6, and CYP3A4 to determine the inhibitory effects of sulfaphenazole, ketoconazole, quinidine, troleandomycin, caffeine, and cimetidine on CYPs to reveal potential drug–herb interactions through a high-throughput, fluorescent, 96-well plate screening assay and a parallel artificial membrane permeability assay (PAMPA) [30]. Recently, CYP3A4 baculosomes were employed in inhibition assays to examine 49 herbal species through high-throughput, fluorometric screening together with Herbochip [31]. Vivid^®^ CYP450 enzyme screening kits and Vivid blue (EOMCC) and green substrates (DBOMF, BOMF) for CYP450 baculosomes (CYP3A4, CYP2C9 and CYP2D6) were used for mechanism-based inhibition by ketoconazole, sulfaphenazole, and quinidine through a fluorescent assay combining enzyme encapsulation techniques, the microarray method, and wide-field imaging [32].

The fluorescence-based, high-throughput approach for the screening of the inhibitory effects of xenobiotic compounds on CYP activities demonstrates numerous advantages as compared to traditional, HPLC-based enzyme assays. It is faster as a shorter period of time is required for sequential data acquisition; it also costs less as costs for reagents are minimized with little loss in data quality. Additionally, it is also particular useful for enzymes with low expression such as polymorphic variants and mechanism-based assays carried out through the dilution method. Nevertheless, caution need to be exercised when applying the fluorescence-based assays. At first, the test compounds should not exhibit fluorescence properties that interfere with the fluorometric measurement of metabolite. Excess NADPH may interfere with fluorometric detection of metabolites such as 3-hydroxy-5,5-dimethyl-4-(4-methylsulfonylphenyl)-(5*H*)-furan-2-one) (DFH), which requires further experimental steps to remove excess NADPH at the end of the incubation [21]. Some of these disadvantages have been resolved by structurally modifying common fluorogenic substrates supplied by Vivid^®^ CYP450 Screening Kits.

## 3. Luminescence-Based Assay

**Figure 3:**
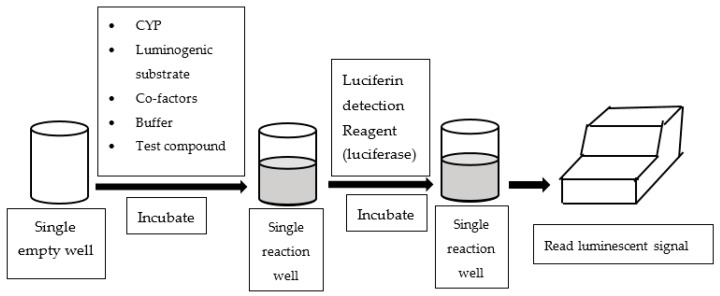
Work flow of luminescence-based assay.

An alternative assay configured in multi-well plates is to measure luminescent readings to quantify CYP enzyme activities. Luminescence-based CYP assays employ derivatives of luciferin as CYP probe substrates, which are luminogenic [33]. In essence, the luminogenic probe substrates are metabolized by CYP to luciferin, which subsequently reacts with luciferase and produces luminescent light (See Figure 3). Most of the luminescence-based CYP assays available in literature utilized P450-Glo^TM^ assay kits supplied by Promaga Corporation (Madison, WI, USA).

P450-Glo^TM^ assay kits are able to investigate the effects of test compounds on the enzyme activity of a CYP of interest. Furthermore, since the P450-Glo^TM^ substrates and metabolites are permeable to cells, numerous cell-based assays employed these kits to examine CYP activities. Mesalazine and mosapride were screened for the induction of CYPs (1A2, 2B6, 2C9, and 3A4) using cryopreserved human hepatocytes [34]. Moreover, CYPs (1A1, 1A2, 1B1 and 3A4) from liver cells employ a tryptophan derivative, 2-(1′H-indole-3′-carbonyl)-thiazole-4-carboxylic acid methyl ester (ITE), as the substrate to test the metabolic function of 2-(1′H-indole-3′-carbonyl)-thiazole-4-carboxylic acid methyl ester (ITE) in *Huh7* and *C3A* cells [35]. HepG2 cell lines expressing CYP enzymes (CYP2C9, CYP2C19, CYP2D6, and CYP3A4) were tested with inhibitors (ethinylestradiol, ritonavir, mifepristone, erythromycin, clarithromycin, roxithromycin, tienillic acid, ticlopidine, and paroxetine) to evaluate hepatic drug metabolism, hepatotoxicity (drug–drug interaction), and mechanism-based inhibition through the assay mentioned above [36]. This approach was also used to test the inductive effects on CYP1A2, CYP2C9, CYP3A4, and CYP1A1 from mesenchymal stem cells (MSC), HepG2, and hepatocyte-like cells by rifampicin and omeprazole employing luciferin CYPspecific substrates [37]. Recently, a newly introduced method, magnetic 3D cell culture with a luminescent assay in 384-well plate (P450-Glo and CellTiter-Glo), was used to examine primary human hepatocytes (CYP3A4, CYP2B6, and CYP1A2) employing luciferin pro-substrates (Promega) for CYP induction and inhibition by verapamil, ticlopidine, and α-napthoflavone [38].

Using a luminescence-based, high-throughput assay was an effective, cheap, and highly sensitive method for enzyme screening and was suitable for CYP screening during early drug discovery, especially for pharmacokinetics and toxicity studies. It is highly flexible in the types of tissue used, sample quantity, isozyme specificity, as well as the method of preparation. This method was rapid and safe as compared to Quantitative Reverse Transcription- Polymerase Chain Reaction (qRT-PCR) methods, and it was also able to reduce the interference between the optical properties of the test compound and CYP substrates [34]. However, the fluorescent assay tends to show readings with higher activity compared to a luminescent assay due to lower concentrations of luminescent substrate used in order to obtain a better luminescent signal-to-noise ratio reading, and this method alone should not be relied on. 

## 4. Mass Spectrometry-Based Assay

**Figure 4:**
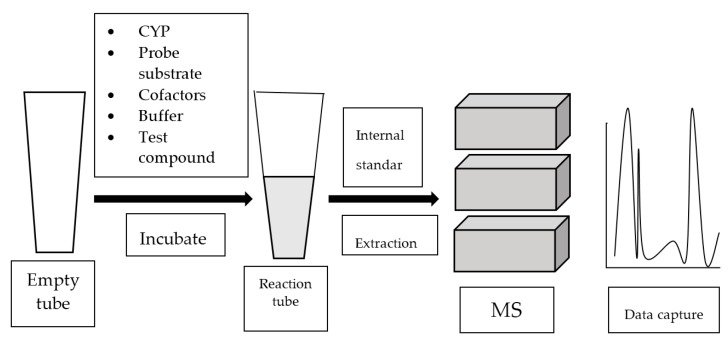
Work flow of mass spectrometry (MS)-based assay.

Mass spectrometry (MS) analyzes gaseous ions via their mass-to-charge ratio (*m*/*z*) [39]. MS systems are usually attached to gas chromatography (GC) or liquid chromatography (LC). Once the individual components in a mixture are ionized, they are separated based on their *m*/*z* by GC or LC. Refer to Figure 4 for the general work flow of MS-based assay.

### 4.1. GC-MS-Based Assay

GC-MS has been applied extensively to quantify and to identify metabolites formed in CYP-catalyzed reactions. It was used to screen baculovirus-infected insect cell microsomes and supersomes containing human cDNA-expressed CYP1A2, CYP2A6, CYP2B6, CYP2C8, CYP2C9, CYP2C19, CYP2D6, CYP2E1, CYP3A4, or CYP3A5, as well as pooled human liver microsomes employing new cathinone-derived designer drugs, 3-bromomethcathinone (3-BMC) and 3-fluoromethcathinone (3-FMC, fluphedrone, flephedrone) to study metabolism activities [40]. GC-MS coupled with the cocktail approach (add multiple substrates in one sitting) was used to screen multiple herb–drug interactions by inhibitors (α-naphthoflavone, 8-methoxypsoralen, sulfaphenazole, *S*-benzylnirvanol, quinidine, diethyldithiocarbamate, and ketoconazole) and the herb, Socheongryong Tang (SCRT), using human liver microsomes CYP2C9, CYP2C19, CYP3A4, CYP1A2, CYP2A6, CYP2D6, and CYP2E1 and employing substrates such as phenacetin, coumarin, cotinine, diclofenac, *S*-mephenytoin, dextromethorphan, chlorzoxazone, and testosterone [41].

Nevertheless, the analyte derivatization process prior to GC-MS is laborious, thus limiting its utility for metabolite identification. On the other hand, LC-MS or LC-MS/MS (liquid chromatography tandem mass spectrometry) has been evidenced to be a more powerful, analytical approach to characterize structures and to quantify drug metabolites in CYP reactions.

### 4.2. LC-MS or LC-MS/MS-Based Assay

CYP1A2 and CYP3A4 from human C3A and HepaRG cells, together with phenacetin and testosterone as the substrates were screened for their phenotypic and metabolic parameters through LC-MS/MS by prototypical inducers (omeprazole and rifampicin) and CYP isoform-specific inhibitors (fluvoxamine and ketoconazole) [42]. Additionally, LC-MS/MS was used to perform metabolic studies using human liver microsomes with a range of substrates (cefotaxime, gemifloxacin, ciprofloxacin, fluconazole, gentamicin, clindamycin, linezolid, and metronidazole) [43]. Similarly, pharmacokinetics analysis targeting CYP1A2, CYP2A, CYP2C19, CYP2D6, CYP2E1, and CYP3A from rat liver microsomes involved the use of the substrates phenacetin, coumarin, omeprazole, dextromethorphan, and chlorzoxazone with the test compound, chrysosplenetin, through the LC-MS/MS method [44]. 

LC-MS/MS system has been further equipped with 96-well microplates to strengthen high-throughput. It was used for functional screening of 14 steroid substrates (testosterone, 17-methyltestosterone, progesterone, pregnenolone, estrone, cortisol, 19-norandrostenedione, dehydroepiandrosterone (DHEA), cortexolone, corticosterone, 17-hydroxy- and 21-hydroxyprogesterone, *cis*-androsterone (3α-hydroxy-5α-androstan-17-one), and trans-androsterone (3β-hydroxy-5α-androstan-17-one) with yeast-expressed, recombinant, mammalian CYP1A enzymes (human CYP1A1 and CYP1A2, mouse CYP1A1 and rabbit CYP1A2) [45]. Moreover, high-throughput was also improved by coupling LC-MS/MS with the cocktail approach, which is also known as n-in-one assays and tested for the inhibitory effects of several CYP isoforms simultaneously. Ultra-performance liquid chromatography (UPLC-MS/MS) with the cocktail method was used to screen CYP2B1, CYP1A2, CYP2C11, CYP2D6, CYP3A4, and CYP2C9 from rats employing bupropion, phenacetin, tolbutamide, metoprolol, testosterone, and omeprazole as the substrates with MGCD0103 in the metabolic study [46]. Another study employed fast gradient LC-MS/MS to examine the inhibition of CYP inhibitors (ketoconazole, quinidine, sulfaphenazole, tranylcypromine, quercetin, furafylline, and 8-methoxypsoralen) on human CYP3A4, CYP2D6, CYP2C9, CYP1A2, CYP2C19, CYP2A6, and CYP2C8 using a cocktail of probe substrates (midazolam, bufuralol, diclofenac, ethoxyresorufin, *S*-mephenytoin, coumarin, and paclitaxel) [47]. Likewise, a high-throughput inhibition screening of human liver microsomes and recombinant CYP2A6, CYP2C9, CYP2C19, CYP2D6, and CYP3A4 involved the use of probe substrates (coumarin, tolbutamide, *S*-mephenytoin, metoprolol, and midazolam) by inhibitors (tranylcypromin, sulfaphenazole, ticlopidine, quinidine, and ketoconazole) through in vitro cocktail and LC-MS/MS [48]. A combination of LC-MS/MS, 96-well plates, as well as the cocktail approach has provided a faster and more effective screening option. It was employed to screen inhibitory potencies of inhibitors (sulfaphenazole, nootkatone, quinidine, quercetin, α-naphthoflavone, ketoconazole) on CYP (1A2, 2C8, 2C9, 2C19, 2D6, and 3A4) from human liver microsomes using the substrates phenacetin, amodiaquine, diclofenac, *S*-mephenytoin, dextromethorphan, and midazolam [49]. It was also seen that LC-MS/MS with a 96-well plate coupled with cocktail substrates (phenacetin, coumarin, bupropion, amodiaquine, diclofenac, *S*-mephenytoin, bufuralol, midazolam, and testosterone) was applied to screen direct inhibition (DI) and time-dependent inhibition (TDI) by test compounds including itraconazole, fluvoxamine, and fluconazole for DI, while furafylline, tienilic acid, ticlopidine, paroxetine, and erythromycin were used for TDI on CYPs from liver microsomes (CYP1A2, CYP2A6, CYP2B6, CYP2C8, CYP2C9, CYP2C19, CYP2D6, and CYP3A4/5) [50]. 

LC-MS/MS is considered to be a fast and highly sensitive analytical approach for the quantification of metabolites. It is able to produce large sets of experimental data and also has the ability to produce data with high accuracy. Furthermore, LC-MS/MS can be coupled with the cocktail method, which allows the determination of multiple CYP activities simultaneously. LC-MS/MS with a cocktail assay is adaptable for automation and small volumes. On the contrary, LC-MS/MS requires electrospray ionization prior to screening, and the equipment is relatively expensive. Additionally, some metabolites are not sensitive enough to be detected by this method. Stable-isotope compounds were required for CYP-specific probe substrate metabolites as an internal standard to avoid interference by ion suppression in LC-MS/MS quantification, and this method was not suitable for initial drug discovery as compared to fluorescent and luminescent assays, which provided the highest throughput [50]. A cocktail assay should be used with concern as interactions might occur among the probe substrates, and the use of high microsomal protein concentration levels might complicate the data interpretation due to the nonspecific binding of inhibitors or substrates to the microsomal protein.

## 5. Conclusions

CYP family of enzymes are important phase I enzymes responsible for drug clearance or the bioactivation of prodrugs. At the early stage of drug discovery and development, in vitro investigations of modulatory effects of new drug entities or herbal preparations on CYP activities play key roles in selecting suitable therapeutic candidates for subsequent in vivo and clinical trials. A high-throughput screening approach optimizes the chance of identifying lead compounds from a large number of agents. Currently, several high-throughput methods including fluorescence-based, luminescence-based, and MS-based assays have been developed and widely applied to quantify CYP activities. Despite fast screening processes, each approach is has certain advantages and disadvantages (see Table 2). It is advised to consider multiple factors such as available equipment in the laboratory, budget, and enzyme sources supplied before making a choice from these methods. It would be ideal to apply more than one approach for the screening of modulatory effects of compounds on CYP activities if possible. In the future, ultra-high-throughput screening methods with an automated system should be further developed and validated to meet the requirement of drug discovery and development in the new era. 

## Figures and Tables

**Table 1:** Common fluorogenic substrates of cytochrome P450 (CYPs).

Substrate	CYP	Metabolite	Ex/Em (nm)
CEC	CYP1A1/CYP1A2/CYP2C19	CHC	408/455
Coumarin	CYP2A6	7-HC	355/460
BFC	CYP3A4	HFC	410/510
EFC	CYP2B6	HFC	410/510
MFC	CYP2C9/CYP2E1/CYP2C19	HFC	410/510
DBF	CYP2C8/CYP3A4/CYP2C9/CYP2C19	Fluorescein	485/538
AMMC	CYP2D6	AHMC	390/460
MAMC	CYP2D6	HAMC	390/460
DFB	CYP3A4	DFH	360/440
EOMCC	CYP1A2/CYP2C19/CYP2D6	CHC	408/455
BOMCC	CYP2C9/CYP3A4	CHC	408/455
BOMF	CYP2C9	Fluorescein	485/538
BQ	CYP3A4	7-hydroxyquinoline	358/505
BzRes	CYP3A4	Fluorescein	485/538
DBOMF	CYP3A4	Fluorescein	485/538

CEC: 3-cyano-7-ethoxycoumarin; BFC: 7-benzyloxy-4-(trifluoromethyl) coumarin; EFC: 7-ethoxytrif luoromethyl coumarin; MFC: 7-methoxy-4-(trifluoromethyl) coumarin; DBF: dibenzylfluorescein; AMMC: 3-[2-(*N*,*N*-diethyl-*N*-methylammonium)ethyl]-7-methoxy-4-methylcoumarin; MAMC: 7-methoxy-4-(aminomethyl) coumarin; DFB: (3,4-difluorobenzyloxy)-5,5-dimethyl-4-(4-methylsulfonylphenyl)-(5H)-furan-2-one); BOMCC: 7-benzyloxymethyloxy-3-cyanocoumarin; DBOMF: dibenzyloxymethylfluore scein; EOMCC: ethoxymethyloxy-3-cyanocoumarin; BOMF: benzyloxy-methyl-fluorescein; BQ: 7-benzyloxyquinoline; BzRes: benzylresorufin; CHC: 3-cyano-hydroxycoumarin; 7-HC: 7-hydroxycoumarin; HFC: 7-hydroxy-4-(trifluoromethyl) coumarin; AHMC: 3-[2-*N*,*N*-diethyl-N-methylammonium)ethyl]-7-methoxy-4-methylcoumarin; HAMC: 7-hydroxy-4-(aminomethyl)-coumarin; DFH: (hydroxy)-4-(4-methylsulfonylphenyl)-5,5-dimethyl-(5H) furan-2-one).

**Table 2:** Major advantages and disadvantages of various high-throughput approaches.

Advantages/Disadvantages	Fluorescence-Based Assay	Luminescence-Based Assay	MS-Based Assay
Advantages	✓ Fast and cheap ✓ Minimized reagent consumption	✓ Fast and cheap ✓ Minimized reagent consumption ✓ Flexible in the types of tissues used	✓ Highly sensitive ✓ Accurate
Disadvantages	Probe substrates may be nonselective, thus heterologous- expressed individual CYP should be used Possible fluorescence interference with metabolite by test compounds	Lower activity reading as compared to fluorescence-based assay	Equipment is relatively expensive Need to use internal standard Less high-throughput than fluorescence/luminescence-based assay
